# Speech and Language Skills of Low-Risk Preterm and Full-Term Late Talkers: The Role of Child Factors and Parent Input

**DOI:** 10.3390/ijerph17207684

**Published:** 2020-10-21

**Authors:** Chiara Suttora, Annalisa Guarini, Mariagrazia Zuccarini, Arianna Aceti, Luigi Corvaglia, Alessandra Sansavini

**Affiliations:** 1 Department of Psychology “Renzo Canestrari”, University of Bologna, 40127 Bologna, Italy; annalisa.guarini@unibo.it (A.G.); mariagrazia.zuccarini@unibo.it (M.Z.); 2 Neonatology and Neonatal Intensive Care Unit, S. Orsola-Malpighi Hospital, 40138 Bologna, Italy; arianna.aceti2@unibo.it (A.A.); luigi.corvaglia@unibo.it (L.C.); 3 Department of Medical and Surgical Sciences, University of Bologna, 40126 Bologna, Italy

**Keywords:** language delay, late talkers, early predictors, preterm birth, child spontaneous speech, MB-CDI, parent–child book sharing, child-directed speech

## Abstract

Among children in the third year of life, late talkers comprise from 9% to 20%. This range seems to increase when addressing preterm children. This study examined video-recorded child spontaneous speech during parent–child book sharing as well as linguistic skills reported through the MacArthur Bates Communicative Development Inventories (MB-CDI) Short Form in 61 late talkers aged 30 months old (26 low-risk preterm, 8 females; 35 full-term, 12 females). Differences between low-risk preterm and full-term late talkers in child language measures and parental speech input were tested, as were the roles of child and parent factors on child language. Low-risk preterm and full-term late talkers showed similar speech and language skills. Similarly, no differences were found in measures of parental speech between groups. Child cognitive score, chronological age, and low-risk preterm status were positively associated with lexical diversity, rate, and composition of child speech production, whereas family history for language and/or learning disorders as well as parent measures of lexical diversity, rate, and grammatical complexity were negatively associated with the above child variables. In addition, child cognitive score and low-risk preterm status were positively associated with the MB-CDI measures of word and sentence production. Findings are discussed in terms of the need of good practices when following up on low-risk preterm children and of interventions targeting parents’ input to preterm and full-term late talkers.

## 1. Introduction

“Late talkers” are defined as children aged 18 to 35 months who exhibit slower language development and a limited expressive vocabulary with respect to their typically developing peers, and who do not have concomitant developmental disabilities, sensory impairments or cognitive or socio-emotional deficits [1,2,3]. Prevalence studies have indicated that children identified as late talkers ranged from 9% to 20% of children aged between 24 and 36 months [4,5,6,7,8]. 

Overall, there was great variability in the prognosis for late talkers. Indeed, many children, defined as “late bloomers,” catch-up in their delay by the age of 3–4 years [9,10]. However, a consistent group of late talkers show persistent language difficulties ranging from 6% to 44% [1]. This fluctuation may be due to sampling inclusion criteria; studies with less strict criteria and more heterogeneous samples showed a greater proportion of children with persistent linguistic difficulties [11], which may have also cascading effects on later academic achievements [3]. 

### 1.1. Indirect and Direct Measures of Late Talkers’ Speech

Identifying late talkers is often based on measures of expressive vocabulary calculated by parental reports, such as the MacArthur-Bates Communicative Development Inventories (MB-CDI) [12]. Based on the MB-CDI, 24- to 36-month-old children are considered late talkers if the number of words included in their expressive vocabulary is equivalent or lower than the 10th percentile [13,14,15]. Results from parental reports describe late talkers as weak in several communicative and linguistic areas besides expressive vocabulary. For instance, using the MB-CDI short form, Bello et al. [13] found significant weaknesses in late talkers’ gesture production, phonological competence, and lexical comprehension. Grammatical development was also delayed in late talkers. Comparing a group of late talkers with a group of toddlers on the autism spectrum at 30 months, based on the MB-CDI, Weismer et al. [16] found that in both groups, more than half of the participants could not yet combine words. The authors also noted a significant association between word production and grammatical complexity scores for late talkers, a result supporting the link between these linguistic domains also in children with language delay.

Although indirect measures of speech and language such as parental reports constitute a very rich and validated material for researching late talkers’ language delays, direct assessment of children’s speech may provide scholars and clinicians with more in-depth analysis of child competences with their crucial measures, such as word types and tokens, and mean length of utterances (MLU). Studies that investigated late talkers’ vocal and linguistic competencies from collected speech samples were mainly conducted on small samples of highly heterogeneous children in terms of the age included [17,18,19,20,21,22]. Most of these early studies addressed mainly the phonological skills of late talkers, pointing out significant delays in their consonant and syllabic repertoires [17,18]. In the same direction, another work [19] found that 2–3-year-old late talkers produced significantly fewer vocalizations and more unintelligible utterances than their matched control children. Recently, Chilosi et al. [9] evaluated late talkers’ language sample in terms of grammatical complexity, showing that 84% of their 27-months-old children showed a primitive language organization, mostly characterized by preverbal or holophrastic speech. As for more complex verbal production, Weismer et al. [22] followed four late talkers from 24 to 35 months of age, reporting MLU scores for 75% of children at the age of three ranging from 1.03 to 2.45, which correspond to stages I–II of grammatical development [23]. In addition, Thal & Tobias [21] and Vigil et al. [24] reported below average MLU for late talkers in their samples, indicating significant differences between them and age-controlled matched children also in the amount of spontaneous word production during parent–child interaction. Finally, Rescorla et al. [25] reported that the late talkers in their sample obtained scores 2.5 standard deviations in MLU below the comparison children and in the index of productive syntax, both of which are measures of grammatical development.

### 1.2. Child and Parent Factors of Risk for Language Delay 

Several studies have investigated child and parent factors thought to contribute in predicting the language delay onset in late talkers, suggesting the need for a multifactorial model [26,27]. Even if these factors are mainly implied in the emergence of language delays, as predictors of late talking status among toddlers [4,7,8], their effects, in accounting for individual differences in the lexical and grammatical competences of children already identified as late talkers, have been scarcely investigated [1,14].

#### 1.2.1. Child Factors 

Biological and medical child factors as well as the domain general cognitive processes underpinning language development should be considered [1,27]. Several studies that investigated the effect of preterm birth on the emergence and progress of language delays showed mixed results. Data from large samples followed longitudinally [4,7] indicated that neither preterm birth nor birthweight was a significant predictor of late-talking status from 24 to 30 months of age. By contrast, other investigations that examined populations of children born preterm compared to full-term children, documented to a certain extent the effect of birthweight and preterm birth on the emergence and progress of language delays, particularly for more immature preterm children. Specifically, at two years of corrected age, 20% of a sample of very preterm children (those with gestational age <33 weeks and birthweight <1600 g) performed below the 10th percentile of the MB-CDI questionnaire’s expressive vocabulary score, with males, perinatal medical conditions (i.e., bronchopulmonary dysplasia) and low maternal education increasing the odds of being at risk of delay [28]. At 30 months, 24% of the very preterm children were at risk for language impairment, being below the 10th percentile of the MB-CDI vocabulary score, and 16% resulted at risk when considering the MLU score; at 42 months, 34% of the children were still at risk considering the MLU score as a criterion [29]. Other evidence of delays in preterm children came from a recent European cohort study of children born from 22 to 32 weeks of gestation [30]; at the age of two years, 40% of the children had a low expressive vocabulary (<10th percentile at the MB-CDI) and 25% were not combining words. Language risk increased for children having mothers with low educational levels and for children that were at high perinatal risk (gestational age <28 weeks, severe neonatal morbidity, or severe congenital anomaly) [30]. However, less convergent findings were found for low-risk preterm children (i.e., those not having brain injuries or severe perinatal complications, that are more frequently associated with lower gestational ages). A large Australian sample [8] revealed that children with low neonatal immaturity but a not appropriate fetal growth rate showed difficulties in the language domain, being almost twice at risk for late language emergence at 24 months. By contrast, a longitudinal study on low-risk preterm children did not find significant differences with respect to full-term matched controls during their second and third year of life [31].

Furthermore, studies that investigated the role of gender in predicting early delays in language development showed mixed results. Zubrick et al. [8] indicated that males are three times more at risk than females to show a late language emergence at 24 months, a finding that was recently corroborated by other studies with 24–30-month-old children [4,6]. However, among late talkers there were no gender differences documented in terms of linguistic profiles [14] and gender did not significantly result in predicting late talkers’ later language outcomes, as was recently observed by a meta-analysis [1]. 

Heritability in the emergence and persistence of language delay and impairment constitutes another factor that gained the literature’s attention. Family history of language disorders—having biological relatives with current or past language delays or impairments—emerged as a predictor of language delay in several studies [4,6,8], but it seems to exert no effect on late talkers’ later prognosis, as indicated by a recent meta-analysis [1]. Thus, corroborating evidence is still needed. 

Finally, although a preserved level of cognitive skills represents one of the criteria for the identification of early language delay, late talkers may also present some weaknesses in the global cognitive domain [32] or in some aspects of it, such as symbolic play [13,33]. Recent data also indicated that, among late talkers, those exhibiting worse linguistic abilities in both expressive and receptive skills also had lower cognitive skills [14].

#### 1.2.2. Parent Factors 

Low levels of family socioeconomic status (SES), a low parental education, and a scarce quality of parent–child communicative interaction have been indicated as potential environmental risks for children’s language delay (for a review, see [2]). 

Some authors have proposed that low socioeconomic level does not constitute a threat for language delay per se. Rather, the conditions and mechanisms characterizing this status can indirectly affect children’s development in this area [34]. In children with typical development, a low family SES is often associated with limited educational options, which in turn can affect the quality of parental interactive and communicative practices, as well as child environment in terms of linguistic input [35,36,37]. Literature addressing these factors argued for a predictive role of both family SES and parental education, but with mixed results [4,5,7,8]. Different studies found that two- and three-year-old children who are more likely to experience delays in the emergence of lexicon come from family environments characterized by low parental levels of education and/or poverty [5,7,38]. However, Zubrick et al. [8] reported that the risk for late language emergence at 24 months was not associated with particular levels of SES or parental education, and therefore suggested that children with lower levels of proximal and distal resources had the same chance to be late talkers at 24 months as children with adequate resources. These latter results are in line with data from a recent large community-based cohort [4] in which variables related to parental education and family SES did not have significant results in predicting children’s late-talking status from 24 to 30 months. By contrast, the quality of parent–child interaction, in terms of the frequency of book sharing and joint reading, and levels of engagement in informal play opportunities, resulted as protective factors since they were negatively associated with late talking. Finally, among late talkers, Desmarais et al.’s cluster analysis [14] documented a homogeneity with regard to SES, and, at the same time, highlighted a trend for maternal level of education to be lower for those late talkers with the weakest language and cognitive abilities.

The quality of parent–child communicative exchanges has thus gained scholars’ interest in analyzing the risk and protective factors of language delay. Studies addressing children with typical language development argued that the quality and amount of language input provided by parents to their young children are relevant to language development in various ways. Specifically, when children are approaching the lexical domain, both the amount of speech directed to them, in terms of rate, and its quality, in terms of lexical diversity, support and foster children’s vocabulary growth [37,39,40]. If these aspects of the linguistic environment are so important for children developing typical communicative and language skills, one can argue that the linguistic environment would be just as important for children with language delay. In this direction, only a few studies have examined the linguistic environment of language-delayed children and how this environment can contribute not only to the emergence of a language delay in toddlers, but also to maintaining or, on the contrary, sustaining their later difficulties. The results in this field are interesting but scant. Some studies focused on the effect of parental conversational contingency and the use of language support strategies. Girolametto and colleagues [41,42] reported significant associations between maternal contingent responsiveness (e.g., use of imitation, interpretations, and expansions of child vocal behaviors) and child’s language productivity, both in Italian and Canadian samples of late talkers aged 23 to 34 months. Maternal contingency resulted to account for differences in late talkers’ linguistic skills. Another report corroborated this finding by comparing the input directed to late talkers and control children: authors observed that parents of children with language delays expanded and responded to their children’s utterances to a lesser extent than parents of children with typical language development [24]. With respect to the structural aspects of maternal verbal input, i.e., rate, lexical diversity, and grammatical complexity, Paul and Elwood [43] noted the absence of significant differences in the characteristics of the input directed to toddlers with typical language development and with language delays, with the exception of one measure, i.e., the difference between parental and child MLU, which was higher in dyads of late-talking children. Similar results were highlighted in more recent studies that reported the lack of significant differences in terms of rate (word tokens), diversity (word types and type/token ratio) and complexity (MLU) in the input directed to children with typical and delayed language development [24,44]. Finally, Girolametto et al. [41] found a negative association between maternal rate of speech and late talkers’ vocabulary size at two years, but observed null associations between children’s linguistic outcomes and other aspects of input diversity and complexity (type/token ratio and MLU). To sum these results, parents of children with language delays seem to be less contingent and responsive during communicative interactions, but it is unclear whether their input diversity, rate, and complexity can support children or hinder their language development.

### 1.3. Study Aims 

The present study is aimed at investigating the linguistic skills of 30-month-old late talkers differing in their birth status (low-risk preterm vs full-term) by examining their spontaneous speech production assessed during parent–child book sharing as well as their lexical and grammatical skills collected through parental reports. Specifically, the study aims are the following.

(a) To explore whether linguistic characteristics of late talkers, for both spontaneous speech production (lexical diversity, rate, composition, and grammatical complexity) and reported lexical and grammatical skills (word and sentence production) vary as a function of birth status, controlling also for child chronological age. Considering the divergent literature findings on speech and language outcomes of low-risk preterm children, no major differences were expected between this group and full-term late talkers.
(b) To investigate whether structural aspects of parent input (lexical diversity, rate, composition, and grammatical complexity) directed to late talkers vary as a function of birth status, also controlling for child chronological age. Since this is the first time that differences between parent input to low-risk preterm and full-term late talkers were investigated, the study can be considered explorative.
(c) To examine the role of child (birth status, gender, family history of language and/or learning disorders, cognitive level, and chronological age) and parent factors (educational level and input diversity, rate, and complexity) in accounting for interindividual differences in late talkers’ spontaneous speech and reported linguistic skills. Based on the literature review, significant effects of child and parent factors in predicting child speech and language outcomes were expected, with a significant role of child factors, such as family history of language and/or learning disorders, child cognitive level and chronological age. Concerning birth status, we did not expect that low-risk preterm status would dramatically impact child speech and language outcomes, as findings from literature addressing this population are rather mixed, particularly for low-risk preterm children. As for parent variables, we expected to find a negative role of low parental education and low quality of parent–child communication exchanges on child speech and language outcomes.

## 2. Materials and Methods 

### 2.1. Participants

Sixty-one late talkers, identified with a language screening, participated in the study (see the Procedure paragraph for details). The language screening targeted low-risk preterm children born before 37 weeks of gestational age (GA) at the S. Orsola-Malpighi Hospital of the University of Bologna, and a group of full-term children born at the same hospital, with comparable socio-demographic characteristics. Children with a history of major cerebral damage and/or congenital malformations, visual, hearing or motor impairments as well as children with severe cognitive deficit or with severe neonatal complications were not targeted by the screening since they had already been taken into care by local health services. Children were included in the screening if they were monolingual (Italian) or mainly exposed to Italian since birth. 

The sample consisted of 61 late talkers—26 low-risk preterm children and 35 full-term children—and their parents. Their biological, medical, and socio-demographic characteristics as well as chi-square and *t*-test group comparisons are reported in Table 1. 

As expected, low-risk preterm and full−term late talkers differed significantly on gestational age and birthweight, but did not show significant differences in most of the other variables, i.e., gender, birth order, suffering from otitis media, having family history of language and/or learning disorders (at least a family member), nursery school attendance and being exposed to other parent input, besides the Italian language. Low-risk preterm late talkers spent significantly more days hospitalized, were more often categorized as small for gestational age at birth, and suffered more significantly from respiratory distress syndrome and hyperbilirubinemia needing phototherapy than their peers born at term. Mothers and fathers of low-risk preterm late talkers were significantly older than parents of full-term late talkers. Parental educational levels and nationality distribution between groups did not show significant differences.

The study met ethical guidelines for human subject protections, including adherence to the legal requirements of Italy, and received formal approval from the Bologna Health Authority’s Independent Ethics Committee (EM 194–2017_ and EM 193–2018_ 76/2013/U/Sper/AOUBo). All parents gave informed written consent for study participation, data analysis, and data publication. 

### 2.2. Procedure

Thirty month-old children that have been identified as late talkers, i.e., with an expressive vocabulary size (word production) at/or below the 10th percentile through the use of the Italian version of the MacArthur Bates Communicative Development Inventories (MB-CDI), Words and Sentences Short-Form [46], filled out online by their parents, were invited at the Developmental Psychology Lab, Department of Psychology, University of Bologna, for a direct assessment of their cognitive and linguistic skills. For children born preterm, age was corrected for weeks of prematurity to take into account their level of neurobiological maturation as done in previous studies (see for instance [28,31]). At the time of the screening, low-risk preterm children had a mean corrected age of 30.47 months (SD = 0.84) and a mean chronological age of 31.91 months (SD = 0.98). The mean chronological age of full-term children was 30.33 months (SD = 1.31). At the time of the direct assessment, low-risk preterm children had a mean corrected age of 31.28 months (SD = 0.99) and a mean chronological age of 32.71 months (SD = 1.13). The mean chronological age of full-term children was 31.01 months (SD = 1.34). The difference between low-risk preterm children’s corrected age and full-term children’s chronological age was not significant at the screening (t = −0.46, *p* = 0.664) or at the direct assessment (t (59) = −0.87, *p* = 0.385), whereas children differed significantly in their chronological age both at the time of screening (t (59) = −5.16, *p* < 0.001), and assessment (*t* (59) = −5.25, *p* < 0.001). 

Cognitive skills were assessed through the Bayley Scales of Infant and Toddler Development, Third Edition (BSID-III) [47,48]. Low-risk preterm and full-term late talkers did not differ significantly on their cognitive scores (low-risk preterm children: M = 87.50, SD = 9.30; full-term children: M = 88.43, SD = 10.06; t (59) = −0.87, *p* = 0.385). 

Child speech was collected during a video-recorded 10-min parent-child shared book reading session. A parent, usually the mother (except for two full-term children for whom the father was only available for participating in the session), was invited to interact with her/his child with two age-appropriate picture books at a child-table with two child-chairs where the parent and the child were seated. Sessions had a mean duration of 9 min and 47 s (SD = 96 s).

### 2.3. Tools

Child word production was assessed with the Italian version of the MB-CDI, Short-Form [46]. Short forms of the MB-CDI are reliable widely used tools for identifying late talkers [5,12,49] and the Italian version, validated on 816 Italian children aged 18 to 36 months [46], has already been adopted in screening programs on 2 to 3-year-old children [13]. In the present study, Section 1 and Section 3 of the Italian MB-CDI Words and Sentences Short Form were used. In Section 1, parents were requested to check off, on a list of 100 words, those spontaneously produced by the child. A score of 1 was given for each word checked. The total number of words produced was computed to assess word production. In Section 3, parents were requested to choose for 12 pairs of sentences, each consisting of one incomplete and one complete sentence, which one better represented their child’s sentence production. The number of incomplete and complete sentences and their sum (total sentences) were computed to evaluate sentence production.

### 2.4. Transcription and Coding

Parent’s speech directed to the child and child speech in the videotaped sessions were transcribed into CHAT format of the Child Language Data Exchange System (CHILDES) [50] by a certified speech therapist blind to the study hypotheses and to child age and birth status. The unit of transcription was the utterance, defined as any speech sound, word or sequence of words delimited by a pause, a change in the conversational turn or a change in the intonation pattern. With respect to child speech, a vocal utterance was transcribed as a word if it resembled an adult word (plausible phonetic shape), it was potentially relevant to the ongoing situation (plausible context of use), and it met at least three of the following four criteria: (a) occurred at least 2 times; (b) was phonetically similar to the target word; (c) had a specific referent; and (d) was recognized by the parent [51]. All children’s vocal utterances not satisfying these criteria were transcribed in IPA and marked as unintelligible speech productions. Children’s onomatopoeic sounds, interjections and repetition of parent’s speech were also marked in the transcriptions. A morpho-syntactic tier in which words were classified by their syntactic category was added to the transcripts using the MOR command under CLAN software.

A researcher (the first author of this manuscript), also blind to the children’s ages and birth status during the transcription, transcribed a randomly assigned 20% of the parent-child sessions to establish transcription reliability. Reliability between the two transcribers was high, with a percent interrater agreement equal to 87% on the segmentation of parents’ utterances and of 85% on the transcription of children’s vocal utterances.

#### 2.4.1. Child Speech

After excluding children’s interjections and repetitions of parent’s speech, children’s productions were analyzed with the CLAN software [50]. The automatized analyses of the transcripts yielded the frequency of: (a) word types; (b) word tokens*;* (c) onomatopoeic tokens*; (*d) unintelligible tokens. The word tokens computation included words from all lexical categories, i.e., nouns (common and proper), verbs, adjectives, and function words (i.e., determiners, pronouns, adverbs, prepositions, conjunctions). A preliminary analysis of the data revealed that approximately 38% of the total word tokens produced by children were the words yes and no*,* i.e., highly routinized verbal productions. To account and control for this result, the frequency of yes/no tokens was calculated, and these tokens were subtracted from the word tokens measure. Furthermore, to assess children’s vocabulary composition the frequencies of nouns, verbs, adjectives, and function words tokens were computed. 

To sum up, the following measures of frequency were calculated for each child participant:

- Word types (the total number of different words produced), as a measure of lexical diversity of child speech;
- Word tokens (all word tokens with the exception of yes and no tokens), as an index of lexical rate of child speech;
- Nouns, verbs, adjectives, and function words*,* as measures of lexical composition of child speech;
- Yes/no tokens*,* as a measure of the rate of highly routinized verbal productions of child speech;
- Onomatopoeic tokens*,* as a measure of the rate of onomatopoeic productions of child speech;
- Unintelligible tokens*,* as a measure of the unintelligibility of child speech.

Raw frequencies were converted into rates per 10 min by dividing the total frequency of each variable by the length of observation in minutes, and then multiplying by 10, to control for the slight differences in the duration of the session among parent-child dyads.

Furthermore, to assess whether children were able or not to produce multi-word utterances, a list of all utterances produced by each child was automatically created by CLAN and total utterances (including only verbal material) per 10 min were computed. A multi-word utterance was defined as a production including at least two different meaningful words in temporal contiguity, and sharing a semantic relation [52]. Children who were able to produce at least two different samples of these utterances were considered competent in the use of word combinations; thus, a dichotomous variable was created, accounting for children who were able or yet unable to combine words. Also, children’s MLU was calculated on the basis of word and verbal utterances (including word tokens and yes/no tokens) produced during the interaction; for four full-term children not showing any word production, MLU score could not be assigned. 

Cohen’s Kappa was computed to verify interrater agreement on child speech coding into verbal, unintelligible or mixed utterances. Interrater reliability was high, with Cohen’s Kappa equal to 0.84. Concerning children’s linguistic outcomes in terms of frequency of word types and word tokens, interrater agreement was achieved by calculating the Intraclass Correlation Coefficients (ICC). Again, the interrater agreement was high, with ICCs > 0.98.

#### 2.4.2. Parent Speech Input 

Parent speech directed to the child was transcribed and analyzed with CLAN software [50]. Onomatopoeic sounds (e.g., “wof”), interjections (e.g., “oh”, “hey”), and incomplete or unintelligible words were excluded from the CLAN analysis. CLAN automated analyses of the transcripts allowed for the calculation of three indexes of quantity and complexity of child-directed speech. The frequency of word types (input types) represented an index of lexical diversity, whereas the frequency of word tokens (input tokens) served as a measure of lexical rate; in addition, the rate of utterances produced (input utterances) was computed. The lexical composition of parents’ verbal input was also examined by computing the frequency of noun (input nouns), verb (input verbs) adjective (input adjective) and function word (input function words) types for each transcript. Grammatical complexity was measured by computing the mean length of utterance (MLU), i.e., the ratio of words to utterance. Raw frequencies were converted in rate per 10 min as done for child speech.

Interrater reliability was calculated using the ICC obtaining high levels of agreement for all parent’s measures (ICCs > 0.95). 

### 2.5. Statistical Analyses

All analyses were carried out using IBM SPSS Statistic 25. All tests were bilateral, and the level of statistical significance was set at 0.05. Data were checked for violation of normality assumption exploring data skewness and kurtosis and using Kolmogorov–Smirnov and Shapiro–Wilk tests. From these analyses, child spontaneous speech data and MB-CDI measures resulted not normally distributed (Kolmogorov–Smirnov and Shapiro–Wilk tests, *ps* < 0.01). To adjust for data non-normal distributions, child speech scores were transformed into ranks. The computed measures of parent’s child directed speech resulted normally distributed.

With regard to the study’s first aim, a set of ANOVAs were carried out to verify the effect of birth status (low-risk preterm vs full-term) on child spontaneous speech measures, in terms of lexical diversity (word types), rate of speech (word tokens, yes/no tokens, onomatopoeic, unintelligible), lexical composition (nouns, verbs, adjectives and function words), utterances’ amount and complexity (number of utterances and MLU), and on MB-CDI child’s lexical and grammar measures *(*word, incomplete sentence, complete sentence and total sentence production). A further set of ANCOVAs were conducted on the same measures to control for children’s chronological age at the time of the observational sessions. As regards child’s ability to produce multi-word utterances, potential differences between groups were investigated with the Chi-square statistic. 

Concerning the second aim, ANOVAs and ANCOVAs were run to verify the effect of birth status (low-risk preterm vs full-term) on parents’ verbal input in terms of lexical diversity (input types), rate (input tokens), composition (input nouns, verbs, adjectives and function words), utterances’ amount and grammatical complexity (input utterances and MLU).

Given the large number of comparison performed in the ANOVAs and ANCOVAs, Bonferroni correction was used to establish the level of statistical significance *p* < 0.003 (i.e., *p* < 0.05 divided by 16 comparisons) for tests on children outcomes and *p* < 0.006 (i.e., *p* < 0.05 divided by 8 comparisons) for tests on parents’ verbal input to minimize Type I error.

With regard to the third aim, multiple linear regression analyses (stepwise method with backward variable selection) were performed to investigate the predictors of child spontaneous speech measures (word types, word tokens, nouns, verbs, adjectives, function words, yes/no tokens, onomatopoeic tokens, unintelligible tokens) and of MB-CDI child’s lexical and grammar measures (word, incomplete sentence, complete sentence and total sentence production). To this end, the more relevant measures, able to describe late talkers’ production at this stage of development, were selected for the analyses. In this sense, children’s MLU and amount of utterances were excluded; MLU was not computed for four children and showed very little variation, whereas amount of utterances was redundant. Child (birth status, gender, family history of language or learning disorders, cognitive score, chronological age) and parent’s variables (educational level and measures of parent’s child directed speech diversity—input types, rate—input tokens, and complexity—input MLU) were entered as independent predictors. To investigate potential predictors of child’s ability to produce multi-word utterances, a binary logistic regression (backward selection method) was conducted, entering the abovementioned set of predictors. 

## 3. Results

### 3.1. Low-Risk Preterm and Full-Term Late Talkers’ Speech and Language Measures

The descriptive statistics for the measures of child spontaneous speech for low-risk preterm and full-term late talkers are reported in Table 2. During the 10-min parent−child shared book reading, late talkers produced, on average, approximately 8 verbal types and 14 verbal tokens, whereas nearly 36 communicative productions were unintelligible or consisted of onomatopoeic sounds. In terms of lexical composition, late talkers produced mainly nouns and such routines as yes/no, whereas verbs and adjectives were still rare. Late talkers’ average MLU was very low as it was barely higher than 1 word per utterance.

The results of the first set of ANOVAs performed on these data revealed a general lack of significant differences between low-risk preterm and full-term late talkers when Bonferroni correction was applied (see Table 2). Indeed, low-risk preterm and full-term late talkers produced similar amounts of word types, word tokens, nouns, verbs, and function words; whereas the use of adjective resulted significantly higher for low-risk preterm late talkers. Verbal utterances production was similar between groups, as were MLU scores and the MB-CDI scores. Between-group differences in the production of spontaneous word combinations during parent-child shared book reading, *χ^2^* (1, *n* = 61) = 6.09, *p* = 0.014, were no longer significant when Bonferroni correction was applied, with only 50% of low-risk preterm (*n* = 13) and 35% of full-term late talkers (*n* = 7) exhibiting the ability to combine words in their spontaneous speech.

Table 2 also summarizes the results of ANCOVAs performed on the same measures by controlling for child chronological age. When this variable was entered in the analyses, low-risk preterm and full-term late talkers were comparable on all of the measures of spontaneous and reported speech.

### 3.2. Parent Child Directed Speech 

The descriptive statistics to measure the lexical diversity (input types), rate (input tokens), and composition (input nouns, verbs, adjectives, and function words), as well as utterance amount and complexity (input utterances and MLU) of parental speech input are reported in Table 3. Overall, parents addressed children with approximately 189 utterances with an average MLU of 3.2, using nearly 200 word types, balanced, in terms of nouns and verbs, and rich of function words.

The results of the ANOVAs performed on these data revealed no significant differences on measures of parent child-directed speech between parents of low-risk preterm and full-term late talkers (see Table 3). A lack of differences on measures of parental speech with respect to child birth status persisted when controlling for child chronological age.

### 3.3. Spontaneous Speech and Language Measures: Child and Parent Predictors

The results of multiple linear regression analyses performed on child spontaneous speech and MB−CDI language measures are reported in Table 4. Child (birth status, gender, family history of language and/or learning disorders, cognitive score, chronological age) and parent’s variables (educational level and measures of child directed speech diversity—input types, rate −−input tokens, and complexity—input MLU) were inserted as predictors. Non−significant predictors are not displayed in Table 4.

Concerning the child variables inserted in the models, the regression analyses indicated that low-risk preterm birth exerted a positive effect on children’s production of adjectives and function words, and on children’s MB-CDI measures of word, incomplete sentence, and total sentence production. Family history of language and/or learning disorders negatively predicted children’s word tokens and adjective production. Male gender predicted higher use of onomatopoeic tokens. Children’s cognitive scores were positively associated with children’s word types, word tokens, nouns, and verbs as well as with the MB-CDI measures of word, incomplete sentence and total sentence production whereas negatively associated with the production of unintelligible tokens. Children’s chronological age was positively associated with most of the child speech variables.

Regarding the parent variables inserted into the models, the majority of the child speech measures were predicted by one of the parental input measures considered, with higher levels of input lexical diversity, rate of speech and grammatical complexity, being associated with lower verbal (word types, word tokens, nouns, and function words) and non-verbal (onomatopoeic tokens and unintelligible tokens) child speech productivity. 

Finally, low-risk preterm birth resulted in the only significant positive predictor, *B* = 1.39, *SE* = 0.58, Exp(B) = 4, for children’s ability to produce multi-word combinations, Nagelkerke *R^2^* = 0.13, χ^2^ (1) = 6.11, *p* = 0.013.

## 4. Discussion

This study examined the linguistic skills of low-risk preterm and full-term late talkers and the characteristics of the linguistic input they receive from their parents during interactions. We were particularly interested in investigating potential differences in late talkers’ lexical and grammatical competences—as evaluated in spontaneous speech production and through the MB-CDI questionnaire—that could be associated with low-risk preterm birth status. Whereas the literature has mainly focused on the impact of severe perinatal conditions on child language development [28,53], the present study was aimed at investigating situations characterized by low perinatal risks, as this population of preterm children is often not included in regular follow-up procedures and is not acknowledged as being at risk of language delays. Specifically, this study contributes to the field with novel results by describing the lexical diversity, rate, composition, and grammatical complexity of late talkers’ spontaneous speech, which have received less attention in the literature than standardized measures of linguistic skills have, and by comparing, for the first time, late talkers with differing birth status. The main findings show similar characteristics in low-risk preterm and full-term late talkers’ spontaneous speech and reported language measures, with low-risk preterm late talkers having a slight advantage with respect to their full-term peers in the use of adjectives when the comparison is based on their corrected age, but not when chronological age is considered. These results were consistent with our hypothesis as no major differences due to low-risk preterm birth were expected.

Similar findings emerged for our second aim, as no significant differences were observed in parents’ verbal input addressed to low-risk preterm and full-term children; parents used similar amounts of word types and tokens, as well as utterances comparable in grammatical complexity.

Another major aim of the study was to comprehend the role played by child and parent factors—that some studies have found to predict children language delay [4,7,8,38]—in accounting for interindividual differences in children’s spontaneous speech and reported language measures. The findings, in accordance with our hypothesis, showed that child cognitive score, chronological age, and to a lesser extent low-risk preterm status were positively associated with lexical diversity, rate and composition of child speech production, whereas family history for language and/or learning disorders as well as parent speech measures of lexical diversity, rate, and grammatical complexity were negatively associated with the above child variables, suggesting that parents of less talkative children have trouble with attuning to their children’s linguistic skills. In addition, child cognitive score and low-risk preterm status were positively associated with the MB-CDI measures of word and sentence production. The parental level of education, however, was not significantly associated with either child spontaneous production or reported language skills, as we expected from previous literature’s findings. These findings will be discussed in terms of their implications for the development of good practices when following up on low-risk preterm children and the need of effective interventions targeting parents’ input to preterm and full-term late talkers.

### 4.1. Spontaneous Speech and Reported Linguistic Skills in Low-Risk Preterm and Full-Term Late Talkers

As described in the introduction, studies investigating late talkers’ spontaneous speech during communicative exchanges with their caregivers have mostly been conducted on small samples of children, ranging widely in chronological age (typically from 24 to 35 months, [17,18,19,20,21,22,54]). 

In this regard, our findings extend and bring new results to those of previous literature, having a larger sample of children, all assessed around 30 months of age, and including both low-risk preterm and full-term late talkers. On average, the children in our study produced almost twice the amount of unintelligible, nonmeaningful tokens than they did word tokens. Thus, they exhibited communicative production rooted—up to a certain extent—in a prelexical stage of language development, mostly characterized by preverbal or holophrastic speech similarly to those in findings by Chilosi et al. [9]. This is in line with previous findings [19] on 18 late talkers aged 24 to 31 months old, who showed 13% intelligible utterances during mother–child interactions, as compared to the 50% produced by their typically developing peers. In terms of verbal productivity, an average of 8 word types and 14 tokens were observed for each child every 10 min, with nouns and function words constituting the more frequent child verbal productions and with limited use of verbs and adjectives. Frequent use of nouns was expected because this lexical class develops in the early stages of language acquisition, whereas the frequent use of functional word tokens could be surprising, as use of this lexical class typically increases at more mature stages and serves grammatical development [12,46,55]. However, note that we refer here to the frequency of the use of tokens in the function word class and not to different types of function words, and that these words can be typical of book-sharing settings such as the one chosen for this study (e.g., children replying with “this” or “that” to their parent asking questions regarding book content). With respect to sentence production, only one-third of the children in the sample exhibited the ability to produce multi-word combinations during parent–child book-sharing, and the mean MLU score was 1.09 words per utterance, ranging from 1 to 1.42. This result is in line with the findings by Weismer and colleagues [22] indicating an average MLU of 1.19 in two-year-old late talkers, but is considerably different from the results reported by Rescorla et al. [56], who observed a mean MLU of 2.46 in their three-year-old late talkers (vs. MLU = 4.12 of their control group). These differences may have several explanations. Firstly, significant changes in expressive vocabulary can occur during the third year of life, as documented by monthly normative values reported by large studies with the MB-CDI [12], so we may also expect an increase of expressive vocabulary and MLU in late talkers from 30 to 36 months of age [54]. Second, in the present study, MLU was computed by including yes and no utterances, which are highly routinized and do not add new information, whereas Rescorla et al. [56] excluded such material in computing MLU. Third, in the present study, the child spontaneous speech was collected during a book-sharing session. Even if this context can be effective in eliciting speech, we cannot exclude that some children, who are not used to sharing books, could have been more productive in other parent–child daily interaction contexts. Regarding the language measures collected with the MB-CDI, the results confirm previous literature: the children exhibited small expressive vocabulary size, even if larger than recently indicated by Bello et al. [13] in an Italian sample of 35 late talkers aged 29 months old, all born at term, exhibiting an expressive vocabulary below the 5th percentile—and very low production of both incomplete and complete sentences.

Regarding the role of birth status, we observed - in accordance with our hypothesis - a general lack of difference in the speech and language outcome of low-risk preterm and full-term late talkers. Low-risk preterm late talkers only showed higher rates of adjectives compared to their full-term peers. These results can be quite unexpected, considering that preterm birth and perinatal sufferance are risk factors for language development. However, the present study is the first to explore the differences and similarities in two populations of late talkers differing by birth status. In addition, only low-risk preterm late talkers were included in the present study. Indeed, our findings appear to bring new evidence to previous findings on low-risk preterm children documenting no significant differences based on birth status. As a recent study has shown, when preterm children are not highly immature and have no severe complications, other individual factors, such as cognitive developmental level, appear to have heavier impacts than gestational age does per se [31]. 

Furthermore, to explore more thoroughly our data, group comparisons were also performed by controlling for child chronological age, but no significant differences emerged as well. The choice of performing this kind of control was motivated by recent literature suggesting that employing both corrected and chronological age criteria could provide more complete information and help clinicians to distinguish children who can be at risk of language delays from those who are more likely to catch up with their initial delays [57]. Moreover, putting aside the considerations on the use of age correction for low-risk preterm children, the inclusion of chronological age as a covariate allowed us to control for slight age differences in all children participating in the study. Relative age effects can be common among children at this stage of language development accounting for relevant differences in speech and language skills [46]. 

### 4.2. Child and Parent Predictors of Children Linguistic Production: Is Parent Input too Complex?

The second main purpose of this study was to examine how a set of child and parent variables, which are considered possible predictors of late talking status, can reflect individual children’s differences in spontaneous speech and reported linguistic measures. 

Concerning child factors, late talkers with a family history of language and/or learning disorders comprised about 18% of the present sample, a percentage in line with some literature findings (23% in Zubrick et al. [8]; 30.2% in Collisson et al. [4]). As expected, family history of language and/or learning disorders predicted lower production of word tokens and adjectives in child spontaneous speech, confirming that it could predispose children to a higher risk of language difficulties. In addition, child cognitive development was considered a potential individual predictor, thus Bayley cognitive scores were included in the regression models. Late talkers with higher cognitive scores were ahead in almost every child speech and language outcome. Even if positive correlations between cognitive and language outcomes are widely acknowledged, studies on late talkers that consider cognitive levels in accounting for their lexical variability are scarce [13,14]. In this regard, Desmarais and colleagues [14] performed a cluster analysis on late talkers’ linguistic and cognitive profiles to shed light on the heterogeneity in the language abilities of toddlers with lexical delays. Among the observed clusters, they identified a specific group of children characterized by very weak language abilities, in both comprehension and production, who also exhibited low cognitive development measures. The authors described this cluster of late talkers as corresponding to a more global developmental delay profile than the other clusters captured by the study. To conclude with individual child factors, our findings showed other minor results, such as a gender effect highlighting a greater production of onomatopoeia in boys than girls. Studies on early lexicon development reveal that children with smaller vocabularies produce mostly social terms, routines, and onomatopoeia [58,59] that can be considered as a bridge between actions and symbols [60]. In this light, our results could point to a slight disadvantage of boys over girls. In addition, positive effects of chronological age were found on most of the child outcomes, as expected from the literature on both typically developing and late-talker children [25]. Furthermore, birth status contributed in explaining interindividual differences in MB-CDI-reported measures of word and sentence production, showing positive effects of low-risk preterm status beyond chronological age. In interpreting these findings, we note that the preterm children screened for this study were characterized by low perinatal risk, as local health services had already taken in charge preterm children with more severe neonatal conditions, who were thus not included in the screening. This could help one make sense of the minor differences favoring low-risk preterm children in our sample and could suggest that further studies should include both low- and high-risk preterm late talkers.

With regard to parent factors, this study examined the role of distal and proximal variables, specifically parental education level and various aspects of parent linguistic input. Quite unexpectedly, low parental education level did not affect child linguistic outcomes. A few studies have reported evidence that low parental education predicts language delay [5,10], but our study failed to find any associations in this direction. However, the present study clearly revealed a significant impact of parental input diversity, rate, and complexity on child speech and language outcomes. From the transcription and analysis of parent–child communicative exchanges, we observed that a higher lexical diversity, rate, and grammatical complexity was associated with lower speech productivity among late talkers, considering both meaningful and unintelligible speech production. A greater amount of linguistic stimulation is thought to foster child lexical improvements in children with typical language development [36,61] but seems to have a negative effect on late talkers’ productivity. At the same time, given the correlational nature of our design, this result can also be read the other way: interactions with linguistically immature and less talkative children can lead parents to compensate in exchanges and fill conversational voids with more words and complex utterances. Findings like those observed in the present study have been reported by Girolametto and colleagues [41], who found a negative correlation between the rate per minute of parent input and child vocabulary size and expressive language age. Similarly, Paul and Elwood [43] indicated that differences in terms of MLU between parents and their late-talker children were significantly greater than those observed in control parent–child dyads; this result was interpreted in terms of a limited ability among parents of late talkers to adjust to their children’s linguistic skills. In addition, Girolametto and colleagues [41,62] performed a similar analysis on the basis of these outcomes. First, they refused the hypothesis that structural aspects— lexical diversity, rate, and grammatical complexity—of parental language stimulation would positively impact child linguistic outcomes, and they embraced the idea that parental responsivity, as an input that is semantically contingent on child production, would favor late talkers’ language development. Second, they proposed the hypothesis of an “idiosyncratic feedback loop,” a vicious circle in which late talkers’ phonological and lexical difficulties negatively impact their parents’ talk to them, which in turn constitutes a further obstacle for their child language improvements. 

Our study brings new evidence to these theses in a larger sample of children, documenting that parents of late talkers failed to adjust to their child communicative competences in both quantitative and qualitative input characteristics, i.e., rate, lexical diversity, and grammatical complexity. In addition, no differences in the inputs directed to low-risk preterm and full-term late talkers were observed, corroborating and extending the findings of a previous study conducted on this topic on preterm and full-term children in their first year of life [63]. Implications for practice are discussed below.

### 4.3. Limitations and Strengths of the Study

Some of the present study’s limitations must be considered. First, the inclusion of only low-risk preterm children does not allow for generalization of the findings to all preterm children. As mentioned above [30,53,64], preterm children who suffered severe perinatal conditions are at higher risk of delayed language development. Thus, considering an additional group of high-risk preterm late talkers would allow researchers to gain a better picture of the relationship between preterm birth and language delay. Second, the present paper investigated late talkers’ spontaneous speech at a single age, which may not shed light on the long-term effects of parental speech input on late talkers’ language development. Future studies addressing this topic longitudinally are urged. However, one strength of this work is that our sample size is quite large and rather homogenous in terms of age of assessment, with respect to previous literature on speech samples collected from late talkers. A further strength is the detailed analysis of speech between children and parents, which has not been investigated frequently in late talkers or preterm children, which provides rich cues and information on children’s linguistic abilities and their linguistic environment. 

### 4.4. Implications for Practice

The implications for practice are twofold. The first one regards follow-up recommendations for low-risk preterm children. This study’s findings, together with evidence from recent literature [65], suggest that preterm children with less severe perinatal conditions may be at risk of delays in terms of different developmental competences such as linguistics skills. In this sense, the criteria for their inclusion in follow-up programs should be evaluated. For instance, recent recommendations from the Spanish Society of Neonatology [66] suggest monitoring neurodevelopment outcomes among all preterm children, including late preterm children, at two, four, and five years. Another practical implication concerns interventions aimed at supporting late talkers’ communicative difficulties. Several parent-implemented language interventions for children with language and developmental delays have been developed in the past decades [67,68,69]. Meta-analyses reviewing these interventions have been performed, which reported positive impacts on child communicative and linguistic skills [70,71]. These programs mostly targeted parent–child communicative skills, working to increase reciprocal turn-taking skills, joint attention and parental verbal contingency and responsiveness. Our findings suggest the need to work to reduce parental speech complexity and encourage parents to address their late-talker children with short, simple, and clear utterances while adapting their lexical diversity, rate, and grammatical complexity to their child speech level.

## 5. Conclusions

The present study provides new insights on late talkers’ spontaneous speech and linguistic skills with a focus on the role of low-risk preterm birth in the phenomenon of late talking, which suggest the relevance of analyzing child spontaneous speech and parental input in book-sharing contexts, besides reported child language measures. Our findings call for greater attention to identifying and following up on low-risk preterm children delayed in language development. In addition, they recommend reflection about follow-up practices, inclusion criteria and the need to consider both corrected and chronological age when addressing the preterm population. 

Furthermore, the present study provides novel contributions regarding the role of child and parent factors. Positive effects of child cognitive level of development, low-risk preterm status, and chronological age, whereas negative effects of family history of language and/or learning disorders as well as parent measures of lexical diversity, rate, and grammatical complexity were found, accounting for individual differences among late talkers’ speech and language skills. Thus, this study highlights the mutual influence between late talkers’ speech and the quality of parent speech input, as well as the need for interventions targeting parent input to preterm and full-term late talkers.

## Figures and Tables

**Table 1:** Biological, medical and socio-demographic characteristics of the low-risk preterm and full-term late talkers and their parents and results of group comparisons.

	Low-Risk Preterm Late Talkers (*n* = 26)	Full-Term Late Talkers (*n* = 35)	χ^2^/*t*	*df*	*p*
*Children’s Characteristics*					
GA (weeks), Mean (SD)	33.90 (2.02)	39.37 (1.28)	12.95	59	<0.001
BW (grams), Mean (SD)	1991.58 (457.57)	3249.00 (530.02)	0.66	59	<0.001
Length of Stay in Hospital (days), Mean (SD)	21.61 (35.80)	2.63 (1.52)	−2.70	59	0.003
Gender (Female), *n* (%)	8 (30.80)	12 (34.30)	0.08	1, 61	0.772
Firstborn, *n* (%)	14 (53.80)	16 (45.70)	2.69	1, 61	0.238
Twins, *n* (%)	15 (57.70)	0 (0)	26.78	1, 61	<0.001
Otitis Media, *n* (%)	1 (3.80)	2 (5.70)	0.11	1, 61	0.739
Family History of Language and/or Learning Disorders (LLD), *n* (%)	5 (19.20)	6 (17.10)	0.04	1, 61	0.834
Nursery School Attendance, *n* (%)	17 (65.40)	27 (77.10)	1.03	1, 61	0.311
Other Parent Input Besides Italian, *n* (%)	4 (15.40)	4 (11.40)	0.17	1, 60	0.683
Caesarean Section, *n* (%)	24 (92.30)	8 (22.90)	28.85	1, 61	<0.001
SGA, *n* (%)	5 (19.20)	1 (2.90)	4.51	1, 61	0.034
IVH I/II, *n* (%)	0 (0)	0 (0)	-		-
MV, *n* (%)	2 (7.70)	0 (0)	2.78	1, 61	0.095
RDS, *n* (%)	14 (53.80)	0 (0)	24.46	1, 61	<0.001
Apnea, *n* (%)	1 (3.80)	0 (0)	1.379	1, 61	0.242
BDP, *n* (%)	1 (3.80)	0 (0)	1.37	1, 61	0.242
Sepsis, *n* (%)	1 (3.80)	0 (0)	1.37	1, 61	0.242
ROP I/II, *n* (%)	0 (0)	0 (0)	-		-
Hyperbilirubinemia with Phototherapy, *n* (%)	17 (65.40)	3 (8.60)	21.85	1, 61	<0.001
*Parents’ Characteristics*					
Mother’s Age (years), Mean (SD)	40.38 (5.26)	37.26 (5.33)	−2.26	58	0.028
Father’s Age (years), Mean (SD)	42.63 (5.49)	39.41 (5.72)	−2.07	51	0.043
Mothers with High Educational Level (>13 years), *n* (%)	14 (53.80)	25 (71.40)	2.00	1, 61	0.157
Fathers with High Educational Level (>13 years), *n* (%)	7 (26.90)	18 (51.40)	3.70	1, 61	0.054
Mother’s Nationality (Italian), *n* (%)	23 (88.50)	32 (91.40)	0.15	1, 61	0.700
Father’s Nationality (Italian), *n* (%)	23 (88.50)	32 (91.40)	0.15	1, 61	0.700

GA: gestational age; BW: birthweight; SGA: small for gestational age, infants with a birthweight < 10th percentile for gestational age; IVH I/II: intra-ventricular hemorrhage originating within the subependymal germinal matrix filling less than 10% (I grade) and 50% (II grade), respectively, of the ventricular area on parasagittal view; MV: mechanical ventilation; RDS: respiratory distress syndrome, acute illness coming on within 4–6 h of delivery, characterized clinically by respiratory rate ≥ 60/min, dyspnea and respiratory distress; Apnea: significant apnea was defined as more than four episodes of apnea/hour or more than two episodes of apnea/hour if ventilation with a bag and mask was required; BPD: bronchopulmonary dysplasia, needing both supplemental oxygen for ≥ 28 days and at 36 weeks of post-conception age; sepsis: presence of a positive blood culture and/or clinical and laboratory signs; ROP I/II: retinopathy of prematurity, vasoproliferative retinopathy resolved without a specific therapy before the presumed date of birth; Hyperbilirubinemia with phototherapy: hyperbilirubinemia needing phototherapy according to the criteria proposed by Gomella [45]. Missing data were present for the following variables: Other Parent Input Besides Italian, *n* = 1; Mother’s Age, *n* = 1; Father’s, *n* = 8. Significant results are highlighted in bold.

**Table 2:** Descriptive data for child spontaneous and reported (MB-CDI Words and Sentences Short Form) speech measures. The table also summarizes the *F*, *p* and eta-squared values (η^2^*)* for the ANOVAs and ANCOVAs comparing the child measures of the low-risk preterm and full-term late talkers.

										ANOVA			ANCOVA		
	All Late Talkers (*n* = 61)			Low-Risk Preterm Late Talkers (*n* = 26)			Full-Term Late Talkers (*n* = 35)			Birth Status			Birth Status Controlled for Chronological Age		
	*M*	*SD*	*Range*	*M*	*SD*	*Range*	*M*	*SD*	*Range*	*F* (1,59)	*p*	η^2^	*F* (2,58)	*p*	η^2^
*Child Spontaneous Speech*															
Word Types	8.24	7.38	0.00−30.51	11.12	7.80	0.81–30.51	6.10	6.34	0.00–28.34	7.80	0.007	0.028	2.21	0.143	0.008
Word Tokens	14.00	15.42	0.00–51.21	19.63	17.30	0.00−51.21	9.82	12.54	0.00−50.40	4.95	0.030	0.019	1.34	0.251	0.005
Nouns	5.97	6.38	0.00−20.85	7.89	6.91	0.00−20.85	4.55	5.64	0.00−20.52	3.39	0.071	0.013	0.69	0.411	0.003
Verbs	1.49	3.16	0.00−19.39	2.20	2.86	0.00−9.25	0.96	3.31	0.00−19.39	6.17	0.016	0.019	1.88	0.176	0.006
Adjectives	1.51	2.65	0.00−15.97	2.60	3.44	0.00−15.97	0.69	1.45	0.00−6.84	10.78	0.002	0.032	3.75	0.058	0.011
Function Words	5.03	7.45	0.00−31.99	6.94	8.66	0.00−31.99	3.61	6.15	0.00−25.20	2.58	0.113	0.010	0.92	0.341	0.003
Yes/No Tokens	8.68	11.76	0.00−69.35	10.24	14.30	0.00−69.35	7.52	9.52	0.00−33.87	1.32	0.255	0.005	0.11	0.738	0.000
Onomatopoeic Tokens	1.47	3.23	0.00−21.77	1.38	2.08	0.00−8.96	1.54	3.90	0.00−21.77	0.63	0.432	0.002	0.01	0.945	0.000
Unintelligible Tokens	35.79	24.75	0.79−125.99	37.44	22.14	1.96−88.85	34.56	26.77	0.79−125.99	0.33	0.569	0.001	0.24	0.626	0.001
Utterances	19.88	18.41	0.00−76.08	26.09	20.26	0.81−76.08	15.27	15.66	0.00−53.64	2.75	0.103	0.009	1.22	0.274	0.004
MLU ^a^	1.09	0.12	1.00−1.42	1.11	0.13	1.00−1.38	1.08	0.12	1.00−1.42	1.19	0.279	0.005	0.12	0.729	0.000
*MB−CDI Measures*															
Word Production	18.49	12.67	2.00−40.00	21.38	12.57	2.00−40.00	16.34	12.49	2.00−40−00	2.24	0.140	0.009	2.35	0.131	0.009
Incomplete Sentences	2.69	3.82	0.00−12.00	3.85	4.33	0.00−12.00	1.83	3.19	0.00−12.00	4.46	0.039	0.016	2.96	0.091	0.010
Complete Sentences	0.15	0.54	0.00−3.00	0.11	0.43	0.00−2.00	0.17	0.43	0.00−3.00	0.02	0.880	0.000	0.02	0.887	0.000
Total Sentences	2.84	4.00	0.00−12.00	3.96	4.45	0.00−12.00	2.00	3.46	0.00−12.00	3.75	0.058	.040	2.13	0.150	0.004

^a^ MLU score was calculated on *n* = 57, as four full-term children did not produce any word during the shared book reading session; in bold the significant results corrected for Bonferroni (*p <* 0.003).

**Table 3:** Descriptive data for parent’s child−directed speech (input) variables. The table also summarizes the *F*, *p* and eta−squared values (η^2^*)* for the ANOVAs and ANCOVAs performed on the parent’s child directed speech (input) measures of the low-risk preterm and full-term late talkers.

										ANOVA			ANCOVA		
	Parents of All Late Talkers (*n* = 61)			Parents of Preterm Late Talkers (*n* = 26)			Parents of Full−Term Late Talkers (*n* = 35)			Birth Status			Birth Status Controlled for Chronological Age		
	*M*	*SD*	*Range*	*M*	*SD*	*Range*	*M*	*SD*	*Range*	*F* (1,59)	*p*	η^2^	*F* (2,58)	*p*	η^2^
*Parent’s Input*															
Types	198.43	47.61	87.00−320.00	204.46	40.44	136.00−289.00	193.94	52.43	87.00−320.00	0.73	0.398	0.001	0.50	0.482	0.000
Tokens	606.39	184.04	159.00−1136.00	623.38	140.03	433.00−918.00	593.77	212.06	159.00−1136.00	0.38	0.539	0.001	0.31	0.583	0.000
Nouns	42.23	12.49	18.00−89.00	44.81	10.05	25.00−69−00	40.31	13.87	18.00−89.00	1.96	0.167	0.003	1.13	0.293	0.001
Verbs	46.23	13.38	22.00−85.00	46.23	13.32	22.00−85.00	46.23	13.63	24.00−73.00	0.00	1.000	0.000	0.43	0.513	0.001
Adjectives	10.88	4.39	1.00−21.00	11.54	3.88	3.00−18.00	10.40	4.73	1.00−21.00	1.01	0.321	0.002	0.33	0.570	0.001
Function Words	73.16	16.81	32.00−118.00	73.81	15.25	47.00−118.00	72.68	18.08	32.00−110.00	0.06	0.799	0.000	0.00	0.983	0.000
Utterances	188.70	49.24	53.95−298.64	196.80	39.16	132.26−283.06	182.68	55.36	53.95−298.64	1.23	0.272	0.001	0.45	0.507	0.000
MLU	3.20	0.43	2.37−4.63	3.17	0.33	2.60−3.88	3.23	0.50	2.37−4.63	0.33	0.568	0.000	0.02	0.874	0.000

**Table 4:** Results of multiple linear regression analyses on child spontaneous and reported (MB-CDI Words and Sentences Short Form) speech measures.

Dependent Variables	Predictors	Standardized β	adj. *R*^2^	*F(df)*	*p*
*Child Spontaneous Speech*					
Word Types	Cognitive Score	0.34 **	0.24	7.38 (3, 57)	<0.001
	Chronological Age	0.43 ***			
	Input Types	−0.23 *			
Word Tokens	Family History of LLD	−0.21 ^†^	0.21	4.90 (4, 56)	0.002
	Cognitive Score	0.29 *			
	Chronological Age	0.35 *			
	Input MLU	−0.28 *			
Nouns	Cognitive Score	0.31 *	0.17	5.16 (3, 57)	0.003
	Chronological Age	0.28 *			
	Input MLU	−0.27 *			
Verbs	Cognitive Score	0.30 *	0.14	6.05 (2, 58)	0.004
	Chronological Age	0.36 **			
Adjectives	Birth Status	0.26 ^†^	0.27	6.69 (4, 56)	<0.001
	Family History of LLD	−0.32 **			
	Cognitive Score	0.21 ^†^			
	Chronological Age	0.24 ^†^			
Function Words	Birth Status	0.28 ^†^	0.09	4.08 (2, 58)	0.022
	Input Tokens	−0.29 *			
Yes/No Tokens	Chronological Age	0.22 ^†^	0.11	4.85 (2, 58)	0.011
	Input Types	−0.32 *			
Onomatopoeic Tokens	Gender	−0.37 **	0.20	6.16 (3, 57)	0.001
	Chronological Age	0.20 ^†^			
	Input Tokens	−0.34 **			
Unintelligible Tokens	Cognitive Score	−0.25 *	0.22	9.70 (2, 58)	<0.001
	Input MLU	−0.40 **			
*MB-CDI Measures*					
Word Production	Birth Status	0.22 ^†^	0.23	9.74 (2, 58)	<0.001
	Cognitive Score	0.46 ***			
Incomplete Sentences	Birth Status	0.28 *	0.14	6.10 (2, 58)	0.004
	Cognitive Score	0.32 **			
Total Sentences	Birth Status	0.26 *	0.13	5.39 (2, 58)	0.007
	Cognitive Score	0.31 *			

Child (birth status, gender, family history of language and/or learning disorders [LLDs], cognitive score, chronological age) and parent variables (educational level, input types, input tokens, and input MLU) were entered in the models as independent predictors. Birth Status: full-term = 0, low-risk preterm = 1; gender: M = 0, F = 1; family history of LLD: no = 0, yes = 1; educational level: ≤13 years = 0, >13 years = 1. *** *p* < 0.001, ** *p* < 0.01, * *p* < 0.05, ^†^ *p <* 0.10. The MB-CDI Complete Sentences model is not displayed because no significant results emerged.
